# SARS-CoV-2 as an inflammatory cardiovascular disease: current knowledge and future challenges

**DOI:** 10.2217/fca-2020-0188

**Published:** 2021-03-19

**Authors:** Kevin S Shah, Mary Elizabeth Hale Hammond, Stavros G Drakos, Jeffrey L Anderson, James C Fang, Kirk U Knowlton, Robin M Shaw

**Affiliations:** ^1^Division of Cardiovascular Medicine, University of Utah School of Medicine, Salt Lake City, UT 84132, USA; ^2^Intermountain Healthcare & University of Utah School of Medicine, Salt Lake City, UT 84107, USA; ^3^Nora Eccles Harrison Cardiovascular Research & Training Institute, University of Utah, Salt Lake City, UT 84112, USA; ^4^Intermountain Medical Center Heart Institute, Salt Lake City, UT. University of Utah School of Medicine, Salt Lake City, UT 84107, USA

**Keywords:** cardiomyopathy, cardiovascular system, COVID-19, myocardial injury, myocarditis

## Abstract

SARS-CoV-2 is responsible for the 2020 global coronavirus disease 2019 (COVID-19) pandemic. In patients with COVID-19, multiple cardiovascular (CV) manifestations have been reported. SARS coronavirus 2 infection can lead to inflammatory CV disease first via takeover of the angiotensin-converting enzyme-2 enzyme as a cell receptor as well as the macrophage activation syndrome in severe illness. We review the CV manifestations of COVID-19 and therapeutics under investigation. We discuss the potential long-term CV sequelae after recovery from COVID-19 and the gaps in knowledge including the pathophysiological links between acute cardiac injury, myocardial inflammation and chronic cardiomyopathy. Future investigational efforts could result in significant diagnostic and therapeutic advances potentially impacting the broader field of chronic heart failure and cardiac recovery.

## Lay abstract

COVID-19 has led to a global pandemic, and many patients infected with this novel virus develop cardiovascular (CV) complications including heart attacks, strokes, heart failure and sudden cardiac death. We will review the pathophysiology behind how a viral infection can place a patient at risk and cause multiple CV diseases. Additionally, we will review our current knowledge regarding treatment for the novel corona virus and long-term risk for patients who recover from COVID-19. At last, we will discuss the future perspective regarding what we can learn about how a virus can cause CV disease and how we can better equip ourselves for future pandemics.

On 6 February 2020, the first reported death in the USA with a confirmed cause due to coronavirus disease 2019 (COVID-19) occurred in Santa Clara County, CA, USA. The autopsy report performed on this patient included a ruptured left ventricle in the setting of a minor component of myocardial inflammation due to SARS-CoV-2 [1]. It has been over seven months since the WHO declared COVID-19 a global pandemic, and there have been over 50,000,000 cases and over 1,200,000 deaths globally as of November 2020 [2]. Severe disease or death predominantly occurs in older patients with comorbidities of obesity, cardiovascular (CV) disease, diabetes mellitus, congestive heart failure, hypertension, chronic kidney disease or cancer [3]. Men are more commonly affected than women. Multiple adverse CV syndromes have been described related to COVID-19 including myocardial injury, cardiomyopathy, heart failure, arrhythmias, cerebrovascular accidents, thromboembolism, vasculitis and sudden cardiac death [4–8]. In this review, we will discuss the pathogenesis, clinical presentation and management of inflammatory CV disease caused by COVID-19. Furthermore, as we enter a ‘third wave’ of the pandemic, we will discuss current understanding of the long-term CV implications after infection with SARS-CoV-2 and how to best follow patients who have recovered from COVID-19.

## Pathogenesis of COVID-19 & effects on the CV system

The process of cell infection by SARS-CoV-2 begins with the spike protein (S-protein) of the virus binding to angiotensin-converting enzyme-2 (ACE2) receptors on the host cell membrane in the nasopharynx and lung parenchyma. The S-protein is cleaved into S1 and S2 polyproteins through interactions with host transmembrane protease serine 2 [9]. Via endocytosis, the virus enters the cell, and the positive-strand RNA is released from its envelope. Once the cell is entered, the next step in the viral life cycle is to begin the replication process by utilizing RNA polymerase. After the viral genome has replicated, it utilizes the Golgi apparatus for assembly. From here, the virus moves to the endoplasmic reticulum in preparation for fusion and release into the cytosol. SARS-CoV-2 predominantly infects alveolar type II pneumocytes, vascular endothelial cells and macrophages in the alveoli of the lung. Infection can lead to diffuse alveolar damage, increased vascular permeability with pulmonary edema and acute respiratory distress syndrome in severe cases [10].

**Figure 1.**
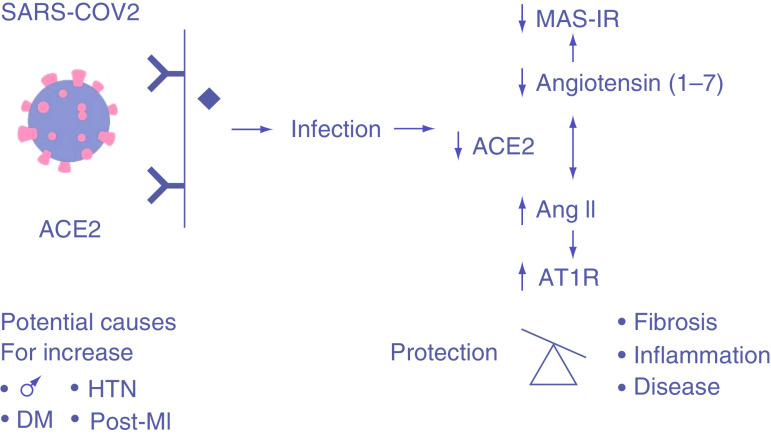
Angiotensin-converting enzyme-2 & SARS coronavirus 2 infection causing downstream pathologic consequences. SARS-CoV-2 utilizes the ACE2 receptor for entry within the cell. After infecting the cell, the downstream consequences of an increase in AT1R lead to an increase in pathologic fibrosis, inflammation and disease.

There are at least three ways that SARS-CoV2 may directly affect the heart and vascular system and either cause disease or aggravate existing CV disease. First, the virus may directly infect myocardial cells or other cells in the heart, causing direct cytopathic effects and tissue destruction. The evidence for this finding is limited, partially due to the few tissue studies performed on patients with COVID-19 [11]. Accumulating evidence from autopsy data suggests that significant myocarditis is rare. By contrast, 48% of autopsy cases showed some thrombi or inflammation within the vessels of the heart suggesting that cytopathic effects on the cardiac vessels are a prevalent mechanism [12]. Second, the virus obtains access to cells by using the ACE2 membrane surface enzyme as a receptor, which has important functional effects in the CV system. Importantly, the virus can infect endothelial cells through the ACE2 receptor and provoke a systemic hypercoagulable state associated with viral-induced vasculitis. Autopsy studies suggest that this is the prevalent mechanism. ACE2, a homolog of ACE, is a monocarboxypeptidase that converts angiotensin II (Ang II) into angiotensin 1–7 (Ang 1–7), which, by virtue of its actions on the endogenous orphan mass receptor (MasR), opposes the molecular and cellular effects of Ang II [13]. The ACE2 receptor is found in many cells in the CV system (endothelium of coronary arteries, myocytes, fibroblasts and epicardial adipocites), with high expression in pericytes. ACE2 is an important renin–angiotensin system regulator capable of mitigating the deleterious actions mediated by Ang II and Ang II type 1 receptor [14]. The renin–angiotensin system has wide-ranging effects on the CV system, including hypertension, heart failure, coronary artery disease and diabetic heart disease. Ang II/Ang II type 1 receptor is critically involved in the disease progression leading to multiple forms of cardiomyopathy and to obesity-associated cardiac dysfunction [15]. By converting Ang II to Ang 1–7, ACE2 shifts the balance to the cardioprotective ACE2/Ang 1–7/MasR axis. With this conversion, pathologic CV consequences including inflammation and fibrosis are catalyzed downstream (Figure 1) [16].

ACE2 is a critical component of the biologic responses to CV insults and the pathophysiologic manifestations of COVID-19. ACE2 is found throughout the CV system, including in the heart, on endothelial cells, smooth muscle cells, macrophages, fibroblasts and pericytes [14]. After myocardial infarction and in those with diabetes mellitus, ACE2 levels are found at greater levels in rats [17,18]. ACE2, through its action to create Ang1–7 from enzymatic action on Ang II, has emerged as a key protective pathway against heart failure (HF). Shedding of membrane-bound ACE2 is likely responsible for the loss of myocardial ACE2 and elevation of plasma ACE2 activity in HF, which correlates with a worse prognosis [16]. ACE2 also is involved with regulation of obesity and epicardial adipose tissue inflammation [15]. In hypertensive patients with diabetes mellitus, serum ACE2 activity is positively correlated with systolic blood pressure [19]. These studies suggest that elevated ACE2 may represent a compensatory or insufficient response to hypertension. SARS-CoV-2 binding to this receptor in the CV system plays a key role in initiating CV dysfunction by its action on ACE2. Kuba *et al.* found that ACE2 expression in the lung was downregulated in mice after SARS-CoV infection or after injection of the SARS-CoV S-protein, leading to increased pulmonary vascular permeability and pulmonary edema [20]. The use of ACE2 to begin the infectious process is the first step in the cascade of events leading to potential downstream CV complications.

### The immune response

**Figure 2.**
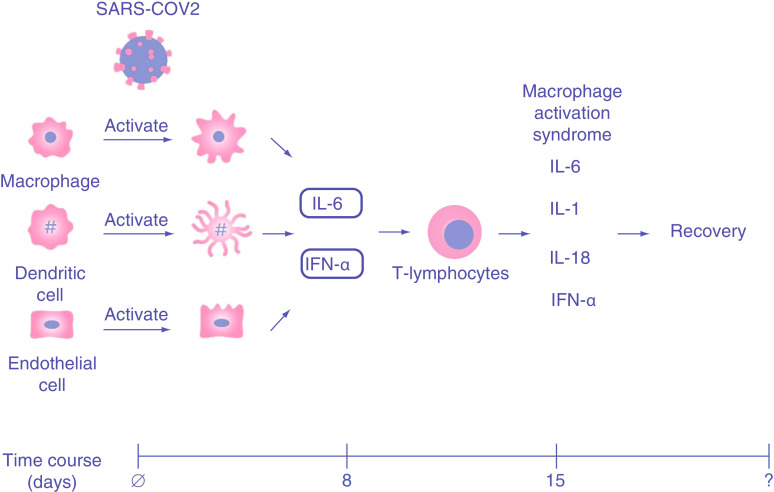
The immune cells are activated by the SARS-CoV-2 leading to cytokines release including IL-6 and IFN-α. These lead to downstream activation of the macrophage activation syndrome, which are responsible for the inflammatory cardiovascular disease with COVID-19.

The third way that the virus can affect the heart and CV system is through immune dysregulation and hyperactivity. Dendritic cells in the lung are activated by exposure to viral antigen or infection. Pathogen-associated molecular pattern molecules induce danger-associated molecular pattern responses, which lead to expression of chemokines, chiefly chemokine ligands 2 and 7. Dendritic cells circulate to draining lymph nodes where they participate in the activation of T cells, which then migrate to the infected site and produce a variety of cytokines including IFN-γ, TNF-α, IL-2, chemokine ligands 9, 10, 11, and cytotoxic molecules granzyme B and perforin [21]. T-cell helper (CD4) and cytotoxic (CD8) subsets are activated with production of cytotoxic molecules granzyme B and perforin and the activation of B lymphocytes, natural killer cells and macrophages. Cytokines bind to endothelial cells, which increase cytokine expression. IFN-γ inhibits viral replication, and chemokines recruit other innate and adaptive immune cells to combat the pathogen. Cytotoxic molecules directly kill infected cells to eliminate the invading virus. Figure 2 demonstrates the typical immune response and time course in severe COVID-19.

With COVID-19, in contrast to many other viral infections, there is an early dramatic reduction in T lymphocytes despite obvious evidence of activation. The mechanism for this lymphopenia is at present unclear but may represent virally induced interferon suppression (antagonism of IFN-ß signaling) and natural killer cell abnormalities. IFN-γ induces dramatic macrophage activation and infiltration, which may cause a second wave of nontype1 interferon pathway cytokine elaboration, including IL-6, IL-1, IL-18, IFN-γ and colony stimulating factor in an attempt to clear the infection. Such a second wave often generates excessive tissue damage, even in the face of effective clearing of the virus in the lung. IL-6, which has prominent proinflammatory functions, is an important mediator of this response [22]. IL-6 signaling in lymphocytes increases the generation of CD-8 positive cells and promotes activated B-cell maturation. Importantly, it can also anchor to nonimmune cells, including endothelial cells, where its actions can include release of cytokines, promoting vascular permeability and recruitment of monocytes and neutrophils. Measurement of inflammatory markers including IL-6 and D-Dimer, have shown an association with more severe COVID-19 cases [23]. These observations suggest that macrophage activation syndrome (MAS) created by the response to the COVID-19 infection may be an important driver of multi-organ damage, including injury to the heart. Such damage due to the release of cytokines is analogous to cardiotoxicity seen in the setting of chimeric antigen receptor T cell therapy [24]. Activated macrophages and endothelial cells express tissue factor on their surfaces, which then activate the extrinsic clotting cascade leading to fibrin accumulation, hyaline membranes in the lung and thrombi in the arterial tree. Neutralizing IgG antibodies form about 14 days after COVID-19 onset. However, the protective role of these antibodies remains unclear. Small studies with short-term follow-up demonstrate that there is early antibody decay after mild infection, potentially suggesting humoral immunity against SARS-CoV-2 may not be long lasting [25]. Table 1 provides a summary of the time course, inflammatory contributors and clinical findings of COVID-19.

**Table 1.** Time course and cells involved with host response in COVID-19.

Phase of infection	Time line	Description	Host response cell types	Host response mediators	Symptoms/abnormal tests	Modulating factors	Outcome
Acute	1–8 days	Virus infects type II pneumocytes	T cells, DC, macrophages, PMN, NK, CD4, CD8 and B cells	Type I IFN	Fever, dyspnea, weakness and cough	Viral load and lung reserve	Acute pneumonia or other organ injury depending on ACE2 receptor density
Persistent	8–15 days	Virus blocks IFN response; PAMPS/DAMPS innate process blocked, activated macrophages, endothelial cells; cytotoxic damage triggers DAMPS	Activated T cells, B cells, macrophages and endothelial cells	CCL2, CCL7, TLR4, TRL7, IFN-γ, TNF, GM-CSF, IL-6, IL-18 and TF	CRP, Ferritin and D-dimer hypoxemia	Viral load and lung reserve	Acute lung failure
Persistent severe	8–15 days	Lymphocyte exhaustion; continued cytokine storm	Activated T cells, B cells, macrophages and endothelial cells	CCL2, CCL7, TLR4, TLR7, IFN-γ, TNF, GM-CSF, IL-6, IL-18 and TF	ARDS, HF, blood clots, rash and stroke	ACE2 density on cells, obesity, CAD, diabetes and heart failure	Death or recovery
Chronic	Days–weeks	Multiorgan damage	Activated T cells, B cells, macrophages and endothelial cells	TF and fibrin	Depends on organ involved	Organ system with comorbid condition determines expression	Chronic illness, recovery and death

ARDS: Acute respiratory distress syndrome; CAD: Coronary artery disease; CCL: Chemokine ligand; DAMPS: Damage-associated molecular pattern molecule; DC: Dendritic cell; GM-CSF: Granulocyte-macrophage colony stimulating factor; HF: Heart failure; IFN: Interferon; NK: Natural killer cell; PAMPS: Pathogen-associated molecular pattern molecule; PMN: Polymorphonuclear leukocyte; TF: Tissue factor; TLR: Toll-like receptor.

### CV disease related to COVID-19

**Figure 3.**
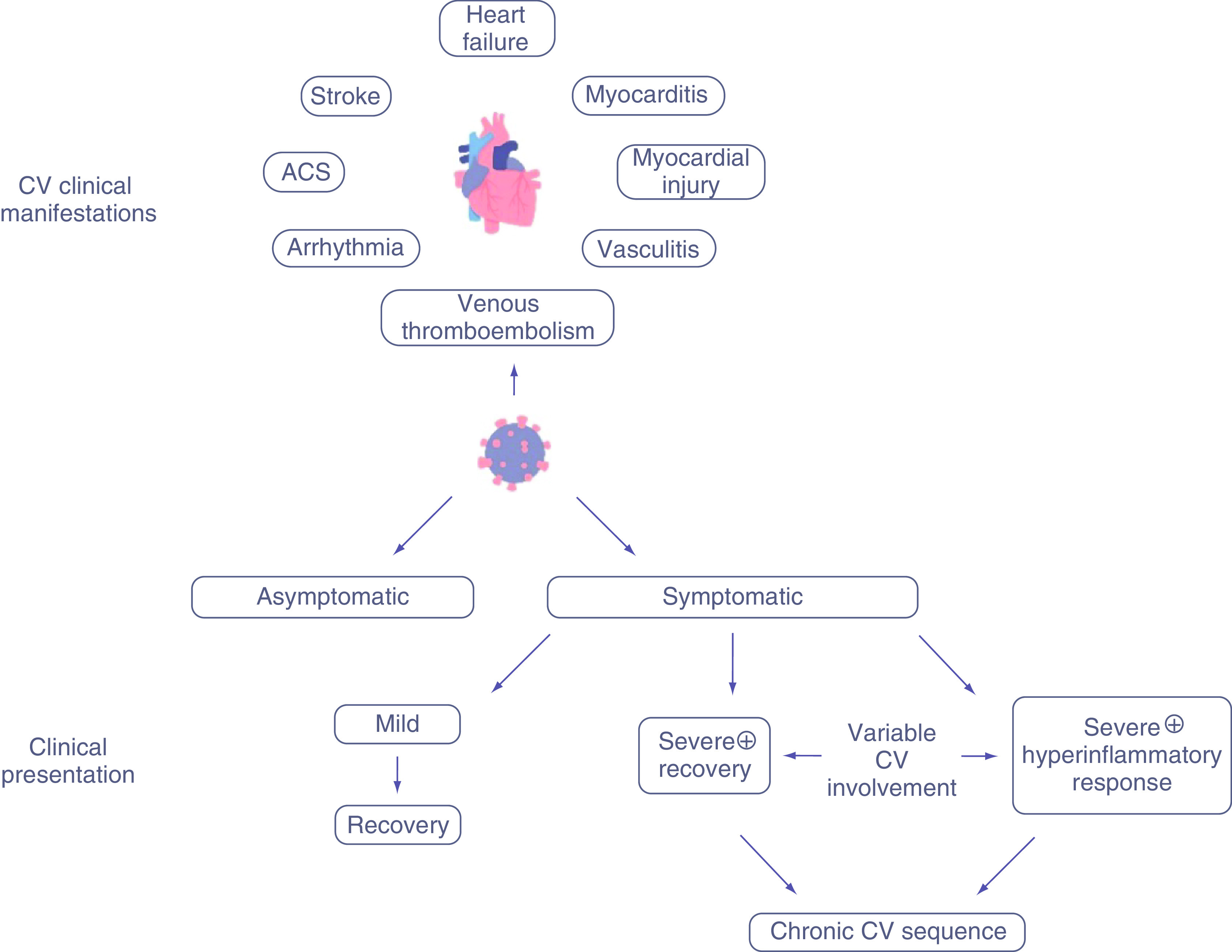
Cardiovascular manifestations of COVID-19. Multiple manifestations have been described and there exist variable presentations in which these may manifest. ACS: Acute coronary syndrome; CV: Cardiovascular.

The clinical presentation of infection from SARS-CoV-2 ranges from asymptomatic to severe respiratory failure with possible CV involvement. Severe illness can occur in otherwise healthy individuals of any age, but more commonly it occurs in adults with advanced age and those with underlying medical comorbidities, particularly obesity [26–28]. Excess ectopic fat deposition and reduced protective cardiorespiratory reserve may potentiate the immune dysregulation seen in COVID-19 critical illness [29]. CV comorbidities, including hypertension, diabetes mellitus and coronary artery disease, are commonly found in those with severe illness requiring hospitalization [30]. CV manifestations of COVID-19 include myocardial injury and dysfunction, arrhythmia, acute coronary syndrome, myocarditis, cardiac tamponade, stress cardiomyopathy, vasculitis, and thromboembolism (Figure 3).

#### Myocardial injury

Large cohort studies from China demonstrated that some patients admitted with COVID-19 had elevated serum troponin and natriuretic peptides, and this finding was associated with adverse outcomes [6,8], including the development of arrhythmias as well as cardiac dysfunction [8]. Among patients who have COVID-19 and underwent echocardiography, cardiac functional abnormalities (wall motion abnormalities, left ventricular dysfunction, diastolic dysfunction, right ventricular dysfunction and pericardial effusions) were present in nearly two-thirds of patients with myocardial injury [31]. Multiple potential mechanisms for cardiac injury have been considered, including myocarditis, acute coronary syndromes, indirect damage due to MAS and vasculitis [32]. Thus, elevated cardiac biomarker levels may be a reflection of myocardial injury related to coronary plaque rupture, reflection of supply/demand mismatch in the setting of hyperinflammatory response, a vasculitis phenomenon or direct myocarditis [32]. Measurement and monitoring of serum troponin may help separate high- versus low-risk individuals admitted with COVID-19 [33,34]. Recent data from Metkus *et al.* have shown that myocardial injury in severe COVID-19 may mostly represent a function of comorbidities and organ dysfunction, similar to what is seen in acute respiratory distress syndrome [35]. This is an important observation, as the finding of myocardial injury continues to have prognostic implications, this is likely related to multisystem organ involvement and critical illness.

#### Arrhythmias

COVID-19 frequently is associated with evidence of CV inflammation, microvascular dysfunction and ischemia, and myocardial injury, which are known precipitants of cardiac arrhythmias [36]. The most significant of these arrhythmias are atrial fibrillation, which involves the atria, and ventricular tachycardia or fibrillation, which involves the ventricles. In one report, atrial fibrillation was among the reasons for transfer to the intensive care unit in 44% of 138 hospitalized patients with COVID-19 [37]. Another cohort of 700 patients with COVID-19 reported a small number of arrhythmias including nine cardiac arrests, 25 atrial fibrillation events, nine clinically significant bradyarrhythmias and ten nonsustained ventricular tachycardias [38]. In another report, Du *et al.* found that among the clinical features of 85 fatal cases of COVID-19, arrhythmias were listed as a complication in 60%, although malignant arrhythmia was given as the cause of death in only two (2.4%) [39]. Our knowledge on the issue of arrhythmias will likely improve with contributions from the COVID-19 registry from the American Heart Association, which is collecting data specifically on arrhythmias [40].

#### Myocarditis

The question as to whether SARS-CoV-2 is directly causing a true viral myocarditis is an area of active investigation. There is evidence that SARS-CoV-2 can infect human pluripotent stem cell-derived cardiomyocytes, leading to inflammation and cell death [41]. Timelines of other viral cardiomyopathies have shown that there is an acute stage as well as possible delayed injury leading to chronic cardiomyopathy. In terms of the novel SARS-CoV-2 coronavirus, there are case reports of a clinical suspicion as well as advanced imaging consistent with myocarditis in patients with COVID-19 [42–45]. In a German study of a patients recovered from COVID-19 infection, cardiac MRI (CMR) revealed cardiac involvement in 78% of patients with ongoing myocardial inflammation in 60% of these patients [46]. A separate study from the USA demonstrated 15% of competitive athletes who were recovering from COVID-19 had evidence of myocarditis on CMR [47]. These studies sparked controversey having providing indirect evidence of ongoing myocardial inflammation in some patients who were recovering from COVID-19; however, their authors acknowledge that the long-term significance of these findings is unclear. A recent literature review of 277 autopsy results in 22 separate publications found that significant myocarditis was found in <2% of autopsies [12]. By contrast, 47.8% of autopsies showed evidence of inflammation and/or fibrin thrombi in the heart vasculature suggesting that this is the source of the inflammatory signals detected on CMR. These features may be related to direct cytopathic action on endothelial cells by the virus or may be indirect consequences of MAS or both. An improved understanding of the frequency of either finding may improve our overall understanding of the pathogenesis of viral myocarditis.

#### Heart failure

Little data exist regarding the development of a new cardiomyopathy or heart failure in the setting of COVID-19. Arentz *et al.* published a case series of patients admitted with COVID-19 to the intensive care unit (ICU) in Washington, and they reported seven of 21 patients (33.3%) developed a cardiomyopathy during hospitalization [48]. This was defined as evidence of globally decreased left ventricular systolic function with signs of cardiogenic shock. Cases of stress-induced cardiomyopathy in the setting of COVID-19 infection have also been reported [49,50]. More recent data from New York demonstrated that a prior history of heart failure was associated with a higher risk of mortality among patients hospitalized for COVID-19 [51]. It is anticipated that as the pandemic progresses and longer follow-up of COVID-19 patients is acquired we will achieve a better understanding of the long-term consequences on the myocardial structure and function and the biological changes that might drive such adverse myocardial remodeling leading to clinical heart failure.

#### Vascular thrombosis & thromboembolism

A major feature and driver of morbidity in hospitalized patients is a hypercoagulable state and subsequent pathology related to clotting. Co-existing with severe illness due to COVID-19 is a pro-inflammatory state with endothelial cell damage that leads to a downstream derangement of hemostasis, potentially triggering both microvascular and macrovascular arterial and venous thrombogenicity [52]. Oxley *et al.* reported five cases from New York of large-vessel stroke in patients younger than 50 [53]. Ackermann *et al.* demonstrated in a small autopsy series widespread thrombosis with microangiopathy on histologic analysis of pulmonary vessels in patients with COVID-19 [54]. In a large Boston registry, a high frequency of major arterial or venous thrombosis and thromboembolism was noted in patients with COVID-19, especially in the intensive care setting and despite thromboprophylaxis [55]. Case series also have been published demonstrating an increased risk of stent thrombosis in patients with COVID-19 [56].

As in the lungs and other organs, microvascular injury/thrombosis in the heart is being recognized increasingly as a cause of myocardial injury [57,58]. Advanced multimodality noninvasive imaging (e.g., echocardiography, positron emission tomography, CMR and CT coronary angiography) can be useful in determining the sites and extent of vascular injury [59].

The prevalence of venous thromboembolism has ranged widely in case series due to the severity of illness. Meta-analyses have demonstrated an incidence of venous thromboembolism ranging from 20–26%, with a higher incidence in critically ill patients [60,61]. In terms of potential therapies, Paranjpe *et al.* demonstrated that among 2773 patients with COVID-19, there was an association of improved hospital survival (both in and out of the intensive care unit setting) in those receiving therapeutic doses of anticoagulation [62]. Ongoing randomized trials will test the hypothesis of anticoagulation to improve outcomes in select inpatients and outpatients [63]. For example, ACTIV-IV is a large multicenter randomized double-blind placebo-controlled trial testing the efficacy and safety of three antithrombotic strategies in COVID-19 adults not requiring hospitalization at time of diagnosis [64]. Regimens include low-dose aspirin, two doses of apixaban (2.5 and 5 mg) and placebo. End points include need for hospitalization and thromboembolic events. The trial was underway as of December 2020. An inpatient trial, testing various parenteral anticoagulant strategies also is underway (NCT 04505774). The long-term management of patients who have developed clotting sequalae related to COVID-19 remains unclear, but is an area of active investigation.

#### Multisystem inflammatory syndrome

SARS-CoV-2 has been described to be integral in the development of a rare hyperinflammatory syndrome with multi-organ involvement similar to Kawasaki disease shock syndrome and toxic shock syndrome [65–68]. In severe cases, patients present with persistent fever, conjunctivitis, edema, gastrointestinal symptoms, extremity pain and coronary artery aneurysms [69]. Belhadjer *et al.* reported a case series of children who experienced cardiac decompensation in the setting of a hyperinflammatory response and offered treatment with immune globulin, which was associated with recovery of left ventricular systolic function [70]. While this manifestation appears rare (as compared with the number of COVID-19 diagnoses), our understanding of the impact of infection within the pediatric population is incomplete and evolving.

### Diagnosis

The diagnostic evaluation of patients with suspected COVID-19 relies on clinical suspicion and confirmatory molecular diagnostic testing. SARS-CoV-2 nucleic acid amplification testing via reverse transcriptase-PCR in symptomatic individuals is recommended in those suspected of having COVID-19 [71]. Specific evaluation for CV manifestations of COVID-19 involves standard selective diagnostic testing including electrocardiography, echocardiography, advanced imaging, blood biomarker measurement and (selectively) heart catherization. A diagnosis of COVID-19-induced cardiomyopathy would be presumptive in the setting of a positive PCR, extracardiac evidence of COVID-19 including inflammatory markers and no obvious primary cardiac disease such as obstructive coronary artery disease. There have not been definitive case series specifically linking COVID-19 to new cardiomyopathy, but there are multiple cases of stress-induced cardiomyopathy in the setting of COVID-19 [72]. The evaluation for myocarditis specifically is typically made by clinical diagnosis with supportive information provided by CMR and/or endomyocardial biopsy [73,74]. Biopsy is often pursued to specifically evaluate fulminant presentations, and exclude rare and potentially treatable causes of myocarditis (e.g., eosinophilic or giant cell myocarditis).

### Therapies

We will briefly review supportive therapies targeted at COVID-19 respiratory illness with attention to known adverse CV effects. There remains a small number (7%) of registered trials specifically planning to evaluate CV therapies [75] as of November 2020. Several potential therapies for COVID-19 not aimed at cardiovascular consequences are being actively investigated and are listed in Table 2.

**Table 2.** Select therapies for COVID-19 under investigation.

Drug	Target	Clinical data	CV adverse effects	Ref.
**Antivirals**				
Hydroxychloroquine	Blockade of viral entry by inhibiting glycosylation of host receptors, proteolytic processing and endosomal acidification	Inconsistent results with multiple trials ongoing	QTc prolongation	[76–79]
Remdesivir	RNA protease inhibitor	RCT failed to show reduction in time to clinical improvement in one trial, NIH trial interim analysis with improved time to recovery	Symptomatic sinus bradycardia	[80,81]
**Immunologic**				
Dexamethasone	Reduction in inflammatory cascade	Randomized trial demonstrating clinical improvement (mortality) in patients requiring oxygen support and mechanical ventilation	Hyperglycemia, hypertension and dyslipidemia	[82]
**Antithrombotics**				
Anticoagulation	Reduce clot burden	Retrospective single center case–control study demonstrating improved survival in those receiving anticoagulation	Bleeding	[62]

CV: Cardiovascular; RCT; Randomized controlled trial; QTc: Corrected QT interval prolongation.

#### Antivirals

Chloroquine and hydroxychloroquine (HCQ) are therapies that have historically been used for several diseases including amebiasis, malaria and lupus. Both agents have immunomodulatory effects through attenuation of cytokine production and inhibition of autophagy and lysosomal activity in host cells. A small RCT had shown the possibility that HCQ may reduce time to clinical recovery [76]. However, experience and contemporary studies have shown that higher doses of chloroquine in critically ill patients may pose risk, specifically QTc interval prolongation [83]. Furthermore, an RCT performed (the RECOVERY trial) showed no mortality benefit in patients hospitalized with COVID-19 randomized to receive HCQ [84]. All subsequently reported therapeutic trials studying HCQ have been negative thus far or have been stopped [85]. Both chloroquine and HCQ can cause QT prolongation; thus, cautious monitoring is important in patients receiving these therapies [86].

Remdesivir is an agent that was discovered through a screening process for antimicrobials, which showed activity against RNA viruses, and was used in the treatment of Ebola. This agent has shown promise against COVID-19 as it has potent *in vitro* activity [87]. Wang *et al.* performed an RCT of 237 patients admitted with severe COVID-19 but reported that it did not demonstrate reduced time to clinical improvement or reduction in viral load [80]. However, the subsequent ACCT-1 trial, which randomized 1062 hospitalized COVID-19 patients to IV remdesivir for 10 days or placebo, demonstrated that therapy reduced the time to recovery from 15 to 10 days (p < 0.001) and led to greater overall clinical improvement at 15 days. Improvement in mortality trended in favor of those receiving remdesivir [88]. In another study, similar findings were noted with a shorter duration of therapy (5-day as compared to 10-day course) [89]. Based on these data, remdesivir has received US FDA approval for use in hospitalized COVID-19 patients.

In contrast to ACTT-1, a preliminary report of the WHO Solidarity trial in 2743 hospitalized COVID-19 patients did not find a beneficial mortality trend (11.0 vs 11.2%) [90]. Heterogeneity among centers in this worldwide trial in implementation and populations has been offered to explain the negative results.

Thus, as with other treatments in COVID-19 (e.g., dexamethasone and monoclonal antibodies), timing and patient selection seem to be important in remdesivir’s benefits. Earlier, outpatient therapy with remdesivir in high-risk patients may hold greater promise and is being tested in an ongoing trial (NCT 04501952).

#### Anti-inflammatories

The use of steroids in hospitalized patients with COVID-19 who require supplemental oxygen has been supported with evidence from the RECOVERY trial [91]. In this study, 2104 patients received dexamethasone as compared with 4321 receiving usual care and there was a short-term mortality benefit in patients who required supplemental oxygen or mechanical ventilation. In a meta-analysis of seven randomized clinical trials with critically ill patients with COVID-19, administration of systemic corticosteroids was associated with lower 28-day all-cause mortality [92]. Adverse CV effects of steroids include hyperglycemia and weight gain.

#### Antithrombotics

Multiple studies have demonstrated a high burden of thromboembolism found in patients with severe COVID-19 [93,94]. Abnormal coagulation parameters are seen in many patients with severe illness, and these abnormalities are associated with worse outcomes [95]. An observational study of 2773 hospitalized COVID-19 patients reported that those that received anticoagulation had an improvement in in-hospital mortality [62]. The International Society of Thrombosis and Haemostasis has developed guidelines to risk stratify patients with COVID-19 for their risk of thrombosis and use of anticoagulation [96]. Future studies are ongoing or being planned to test antithrombotic therapy in a randomized fashion to evaluate benefit and bleeding risk.

#### Vaccines

Critical to ending the pandemic is the development of effective and safe vaccines. Various candidate vaccines are being developed and tested, including nucleic acid vaccines, inactivated virus vaccines, live attenuated vaccines, protein or peptide subunit vaccines and viral-vectored vaccines [97]. Of over 100 proposed vaccines worldwide, at least nine had advanced to Phase III trials as of this writing (December 2020). The two leading candidate vaccines, Pfizer-BioNtech and Moderna, had completed Phase III trials and are on the verge of regulatory approval and wide-spread application. Four leading vaccine candidates for western-world immunization are summarized below.

The Pfizer-BioNtech vaccine is a lipid nanoparticle nucleoside-modified RNA vaccine that encodes the full-length S-protein modified by two proline mutations to lock the host-transcribed viral protein into a prefusion formation and mimic the intact viral protein with which viral-neutralizing antibodies must interact [98]. Phase I/II trials indicated excellent neutralizing antibody induction with mostly mild side effects (e.g., injection site pain, fatigue, headache, chills, muscle pain and joint pain). In the pivotal, 44,000 Phase III trial, 95% effectiveness for protection against COVID-19 was reported without additional safety concerns. The UK medicines regulatory authority approved the vaccine for emergency use 2 December, and population vaccinations began 8 December 2020. Drawbacks include the need for storage at cold temperatures (-70 °C) and a booster dose at 3 weeks. US regulatory review is due 10 December.

Moderna in collaboration with the US National Institutes of Health independently developed an mRNA-based vaccine, which consisted of a modified (sequence-optimized) mRNA encoding the spike glycoprotein encapsulated in lipid nanoparticles. As with Pfizer-BioNtech, Phase I/II trials, early testing of the Moderna vaccine indicated excellent induction of neutralizing antibodies and a similar, acceptable side effect profile [99]. Moderna’s Phase III trial in 30,000 participants also was reported to provide 95% protection, including against severe (hospitalized) cases, without additional safety concerns. Need for a booster dose at 4 weeks is a drawback. FDA regulatory review is due on 17 December 2020.

(Oxford University, UK) and (AstraZeneca, UK) developed a chimpanzee adenovirus-vector delivered vaccine encoding the SARS-CoV-2 spike glycoprotein. Initial trials indicated satisfactory induction of antiviral neutralizing antibodies. The Phase I/II trial was briefly paused after a participant developed neurological symptoms, later linked to multiple sclerosis. Anomalies in the two-dosing regimens associated with a range of outcomes (60–90% efficacy) in the Phase III trial in 30,000 have raised questions to be answered prior to approval and application.

Johnson and Johnson (Janssen Pharmaceuticals, Beerse, Belgium) has launched a Phase III trial in 60,000 participants of a replication-defective adenovirus that expresses full-length spike glycoprotein. A potential advantage is that, unlike other leading vaccine candidates, only a single intramuscular injection, without a booster dose, induces strong neutralizing antibody responses in rhesus macaques and is used in ongoing clinical trials.

Anticipating increasing vaccine availability in early 2021, local and national agencies have been working to prioritize immunization schedules [100]. A general consensus is that front-line healthcare workers and those residing and working in elderly congregant housing (e.g., nursing homes) will constitute tier one, followed by other older and high-risk adults, then other higher-risk population subsets, and finally broad application, with population-wide application estimated to near completion by mid-2021.

### Prognosis & recovery

As we embark upon growing number of cases of COVID-19 within the US, there now exist enough patients who have recovered from infection have listed a number of lingering CV limitations, with the most common symptoms including fatigue and dyspnea [101]. It is not entirely clear how many of these patients’ symptoms can be specifically attributed to CV complications from COVID-19. In some cities, the development of COVID-19 multidisciplinary ‘recovery’ clinics has begun to grow [102]. The long-term recommended follow-up for patients from a CV perspective is largely dependent upon the initial cardiac manifestations. Patients with evidence of myocarditis and myocardial injury will likely need regularly clinical follow-up to determine if these findings progress in to worsening clinical syndromes. Remote rhythm monitoring with telemetry devices for arrhythmias as well as QT interval prolongation will likely be utilized in select patients [103]. A key outstanding question remains as to whether patients who experience myocarditis related to COVID-19 will develop a future cardiomyopathy and clinical heart failure.

### Gaps in knowledge

The exact mechanisms at play to explain the susceptibility of some patients to severe disease and multi-organ involvement remain unclear. Risk prediction of patients who will develop cardiac sequelae from severe COVID-19 is an area of active investigation. It is possible that novel biomarkers of injury and wall stress such as cardiac troponin and natriuretic peptides may reveal patient susceptibility to adverse cardiac events. Furthermore, markers that quantify subcellular myocardial remodeling such as cBIN1 score [104,105], may provide early detection of myocardial changes as well as track and prognosticate clinical outcomes.

The COVID-19 pandemic will provide the opportunity to investigate the interplay between immune dysregulation, myocardial inflammation and myocardial injury, and their impact on the health of cardiomyocytes. Furthermore, the quantification of MAS that occurs in cases of COVID-19 may help delineate the time course and future development of inflammatory CV disease. The COVID-19 pandemic has presented the opportunity to revisit the conversation regarding the contemporary definition of myocarditis. With multiple examples of inflammation of the myocardium (which may not be related to direct viral invasion) as compared with the histologic Dallas criteria to define viral myocarditis, there is contention within the field regarding how appropriate the original criteria are [106]. Perhaps a revised nomenclature distinguishing Dallas criteria myocarditis as compared with less severe myocarditis (which may be secondary to MAS) may be more specific to the findings that are being described globally. Preliminary observations in COVID-19 patients, as well as evidence from other viral causes of cardiac injury, suggest that a primary myocardial injury, and a secondary inflammatory response, are playing key roles in driving the observed CV phenotype.

## Conclusion

The COVID-19 pandemic continues to cause significant morbidity and mortality on a global scale. Patients infected with SARS-CoV-2 are at risk for a wide range of cardiovascular complications, partially related to the body’s own immune response to the virus. Therapeutics for cases of severe COVID-19 have improved over time and with multiple vaccines being approved and launched, there remains hope for the potential to mitigate the COVID-19 over time. Over time and on-going monitoring, we will learn both the short and long-term impact of COVID-19 on the cardiovascular system

## Future perspective

We will continue to make progress in our disease understanding through the COVID-19 pandemic as therapeutics improves and an efficacious vaccine becomes available to the public [107]. We will have an improved understanding in the future regarding which patients are at risk for CV complications after infection with a virus that typically causes a respiratory illness. Furthermore, we will have developed targeted therapeutics specifically aimed at reducing the risk of CV disease after viral infection. We will gain a greater understanding of how the worldwide surge of COVID-19 cases created an unprecedented natural experimental opportunity which could fuel carefully designed studies that could advance our understanding regarding the pathophysiological links between acute myocarditis and dilated cardiomyopathy (viral, idiopathic and other). Such investigational efforts could result in significant diagnostic and therapeutic advances potentially impacting the broader epidemic of chronic heart failure.

**Executive summary**

- The pathogenesis of COVID-19 is related to the novel corona virus SARS coronavirus 2 and cases of infection range from asymptomatic to severe with cardiovascular (CV) consequences.
  - The pathogenesis involves the viral spike protein binding to ACE2 receptors on the host cell membrane, cell entry via endocytosis and subsequent replication.
  - The macrophage activation syndrome in some cases is partially responsible for severe illness as well as CV complications related to COVID-19.
- The CV clinical presentations of COVID-19 are highly variable.
  - Presentations can include new or worsening established heart failure, possible cardiomyopathy, myocarditis, myocardial injury, vasculitis, thromboembolism, arrhythmia, acute coronary syndrome and cerebrovascular accidents.
- The diagnosis of COVID-19 relies on clinical suspicion and molecular diagnostic testing, typically via real-time reverse transcription PCR testing.
  - Evaluation for CV manifestations of COVID-19 involves testing including electrocardiography, echocardiography, advanced imaging, blood biomarker measurement and (selectively) heart catherization.
- The majority of therapeutics remains in development for reducing morbidity and mortality from the viral respiratory syndrome while a small percentage of drugs are being studied specifically to improve CV outcomes.
- Patients who recover from COVID-19 will need long-term follow-up to monitor for potential downstream consequences and a future greater potential burden of CV illness.
- With the potential for widespread vaccination available in 2021, long-term monitoring of those prior infected with COVID-19 will be critical to help understand the interaction between prior viral infection and *de novo* CV disease.
